# Human Decidua-Derived Mesenchymal Stem Cells Differentiate into Functional Alveolar Type II-Like Cells that Synthesize and Secrete Pulmonary Surfactant Complexes

**DOI:** 10.1371/journal.pone.0110195

**Published:** 2014-10-15

**Authors:** Alejandro Cerrada, Paz de la Torre, Jesús Grande, Thomas Haller, Ana I. Flores, Jesús Pérez-Gil

**Affiliations:** 1 Departmento de Bioquímica y Biología Molecular, Facultad de Biología, Universidad Complutense, Madrid, Spain; 2 Instituto de Investigación Hospital 12 de Octubre, Madrid, Spain; 3 Departmento de Obstetricia y Ginecología, Hospital 12 de Octubre, Madrid, Spain; 4 Department of Physiology, Innsbruck Medical University, Innsbruck, Austria; University of Waterloo, Canada

## Abstract

Lung alveolar type II (ATII) cells are specialized in the synthesis and secretion of pulmonary surfactant, a lipid-protein complex that reduces surface tension to minimize the work of breathing. Surfactant synthesis, assembly and secretion are closely regulated and its impairment is associated with severe respiratory disorders. At present, well-established ATII cell culture models are not available. In this work, Decidua-derived Mesenchymal Stem Cells (DMSCs) have been differentiated into Alveolar Type II- Like Cells (ATII-LCs), which display membranous cytoplasmic organelles resembling lamellar bodies, the organelles involved in surfactant storage and secretion by native ATII cells, and accumulate disaturated phospholipid species, a surfactant hallmark. Expression of characteristic ATII cells markers was demonstrated in ATII-LCs at gene and protein level. Mimicking the response of ATII cells to secretagogues, ATII-LCs were able to exocytose lipid-rich assemblies, which displayed highly surface active capabilities, including faster interfacial adsorption kinetics than standard native surfactant, even in the presence of inhibitory agents. ATII-LCs could constitute a highly useful *ex vivo* model for the study of surfactant biogenesis and the mechanisms involved in protein processing and lipid trafficking, as well as the packing and storage of surfactant complexes.

## Introduction

Alveoli, the terminal functional units of the lung's respiratory zone, consist of an endoderm-derived epithelium housing two specialized cell types, the type I and type II pneumocytes. Type II pneumocytes, also named alveolar type II (ATII) cells constitute 5% of the alveolar epithelial surface area and are involved in proliferation and transdifferentiaton into a type I phenotype in response to alveolar damage [1]–[3]. Their main function however is the synthesis and secretion of a surface-active material known as pulmonary surfactant [4]. Surfactant is a complex mixture of lipids and specific proteins that is essential to stabilize the respiratory surface by reducing the surface tension at the air-liquid interface of the thin layer of liquid covering the alveoli. Lung surfactant also participates in innate defense mechanisms [5]–[8]. Lack or impairment of the surfactant system is associated with some severe diseases such as respiratory distress syndrome in infants or acute respiratory distress in children and adults [9]. Much research has been carried out to study pulmonary surfactant biogenesis and secretion by primary cultures of isolated ATII cells purified mainly from mammalian lungs. However, this method is laborious, expensive, animal intensive and has a low yield of cells [10]. Thus, it does not necessarily conform with the 3-R principle of refining, reducing and replacing animal testing as defined by the Basler declaration. In addition, primary ATII cells in culture rapidly lose their original phenotypical features, differentiate into type I-like cell types and fail to proliferate further, precluding their use for long term studies [11], [12]. On the other hand, commercially available cell lines like the L2 and the A549 are inherently problematic as their origin and state of differentiation are ill defined, and no study exists to unambiguously demonstrate that these lines release functionally active surfactant components after physiological stimulations. Cells isolated from the transgenic immortal mouse models (H-2K^b^-tsA58; 10) circumvent the problem of short-term culture to a certain extent, but are still depending on the use of laboratory animals. Thus, surfactant-producing pneumocyte-like cell lines are still not well established, preventing a proper and detailed characterization of biosynthetic and trafficking pathways involved in surfactant biogenesis. In this context, new strategies for obtaining *in vitro* ATII cell cultures have to be taken into account.

In the latest years, stem cells have been extensively investigated as a potential source of alveolar epithelial cells [13]. Murine [14]–[21] and human [20], [22], [23] embryonic stem cells, adult bone marrow-derived stem cells from rat [24] and human [25], [26] as well as human stem cells derived from amniotic fluid [27], amnion [28] or umbilical cord blood [29] have been derivated into cells with phenotypical features consistent with ATII cells. We have previously described a population of mesenchymal stem cells (MSCs) isolated from human extraembryonic membranes termed Decidua-derived Mesenchymal Stem Cells (DMSCs) with the capacity of differentiation into ATII-like cells [30]. To our knowledge, this was the first report on the differentiation capacity of human placenta-derived MSCs into ATII-cells. Placental tissue exhibits certain advantages as a source of MSCs such as an easy isolation of cells without invasive procedures, improbability of viral infection and absence of ethical problems [31], [32]. DMSCs are from maternal origin and display high plasticity, differentiating into derivatives of all germ layers [30], [31], [33].

In the present study, we extend the characterization of differentiation of DMSCs into Alveolar Type II-Like Cells (ATII-LCs) in many aspects as compared to our first report (30). We evaluated the capability of ATII-LCs to express genes encoding surfactant markers and whether those genes are translated into proteins, as well as the ability of ATII-LCs to generate surfactant-like assemblies, to accumulate them in specialized storage organelles and to secrete them upon a proper stimulation. Finally, we determined the biophysical properties of the secreted material exploring its potential as a functional surface active agent. Our results, in particular those related to functional aspects, suggest that the ATII-LCs can be regarded as a novel and very promising model to study intrinsic ATII cell characteristics and functions.

## Experimental Procedures

### Isolation and culture of DMSCs

Human placentas were provided at the Department of Obstetrics and Gynecology from healthy mothers under written consent approved by the Ethics Committee from Hospital Universitario 12 de Octubre. DMSCs were isolated and cultured from extraembryonic membranes as described previously [30]. Briefly, placental tissue was digested with trypsin-versene (Lonza, Spain), cells were seeded at 1.16×10^5^ cells/cm^2^ and cultured at 37°C, 5% CO_2_ and 95% humidity in Dulbecco's modified Eagle Medium (Lonza) supplemented with 2 mM L-glutamine, 0.1 mM sodium pyruvate, 55 µM of B-mercaptoethanol, 1% non-essential amino acids, 1% penicillin/streptomycin, 10% fetal bovine serum and 10 ng/ml of Epidermal Growth Factor (Sigma-Aldrich Química, Spain). Non-adherent cells were eliminated by washing off and adherent cells were grown to confluence and passaged at a density of 4–5×10^4^ cells/cm^2^.

### Differentiation of DMSCs into ATII-LC

DMSCs grown at confluence were differentiated in Small Airway Epithelial Cell Growth Medium bullet kit (Lonza) for 4–5 days. Details on the composition of this medium can be found elsewhere ( http://bio.lonza.com/uploads/tx_mwaxmarketingmaterial/Lonza_ManualsProductInstructions_Instructions_-_Airway_Epithelial_Cell_Systems.pdf ).

By using phase-contrast microscopy, morphological changes in the differentiated cells were examined under a Leica DMIL microscope (Leica Microsistemas SLU, Barcelona, Spain).

### Lipidomic analysis of the PC fraction in DMSCs and ATII-LCs

The molecular species of the phophatidylcoline (PC) fraction of the different whole cell samples were analyzed quantitatively by HPLC coupled to mass spectrometry [34].

### Surfactant harvested from ATII-LCs

Pulmonary surfactant-like complexes were collected from the supernatants of 4 day-differentiated ATII-LCs, stimulated for 6 h at 37°C with ATP (100 µM) and PMA (phorbol 12-mirystate 13-acetate) (100 nM) in buffered solutions containing NaCl 140 mM, KCl 5 mM, MgCl_2_ 1 mM, CaCl_2_ 2 mM and HEPES 10 mM (pH 7.4), supplemented with antibiotics as previously described [35]. Supernatants of DMSCs, treated in the same way, were also collected and served as negative controls. After collection, supernatants were aliquoted and stored at −20°C until use. Before use, supernatants were concentrated by ultracentrifugation at 100.000 g and phospholipid content was determined by phosphorus mineralization [36]. Surfactant from porcine bronchoalveolar lavages, used as a reference, was purified and separated from blood components by NaBr density-gradient centrifugation [37].

### Electrophoresis and Western blot analysis

DMSCs and 4 day-differentiated ATII-LCs were incubated with lysis buffer plus 10% protease inhibitor (Sigma-Aldrich Quimica SA, Madrid, Spain) for 30 min at 4°C with orbital shaking. After centrifugation, clear lysates were collected and aliquoted. Extracellular medium of the cultured cells was also collected and concentrated by centrifugation with a Centripep system (Millipore Iberica SAU, Madrid, Spain) at 3000 g and 25°C. Total protein content was determined by using the Bradford kit (Bio-Rad Laboratories SA., Alcobendas, España). To analyze the content of surfactant proteins, SDS-PAGE was carried out using 10% acrylamide (for SP-A and SP-D) and 16% acrylamide (for SP-B and SP-C) gels under reducing conditions in the presence of 5% β-mercaptoethanol. Proteins were transferred to PVDF membranes using a semidry transfer system at 280 mA for 1 h. Blocking was performed by incubation with PBS/Casein blocker (Bio-Rad Laboratories SA., Alcobendas, España) for 1 h at room temperature. Membranes were incubated with primary antibodies at 4°C overnight with gentle orbital shaking. The primary antibodies used were: rabbit anti-SP-A (1∶500) (Santa Cruz Biotechnology, Inc., Heidelberg, Germany) and rabbit anti-SP-B and anti-SP-C (1∶1000) and mouse anti-SP-D (1∶1000) all from Seven Hills Bioreagents (Cincinnati, OH, USA). After washing, membranes were incubated with secondary antibodies for 1 h at RT. Secondary antibodies were swine anti-rabbit HRP conjugated (1∶1000 for anti-SP-A and 1∶2000 for anti-SP-B and anti-SP-C; Dako Diagnósticos, S.A., Sant Cugat del Vallés, Barcelona, Spain) and anti-mouse peroxidase conjugated (1∶5000; Cell Signalling Technology, IZASA, S. A., L'Hospitalet de Llobregat, Barcelona, Spain). Anti-α-tubulin (1∶10000; Cell Signaling) was used as loading control.

### Quantitative real-time PCR

Total RNA extraction was performed using RNeasy mini kit (Qiagen, IZASA Distribuciones Tecnicas SA, Madrid, Spain). To reverse transcribe the RNA, we used the Transcriptor High Fidelity cDNA Synthesis kit (Roche, Roche España, Madrid, Spain). Real-time PCR was carried out using an ABI 7,500 sequence detection system (Applied Biosystems, Life Technologies SA, Madrid, Spain) with inventoried Taqman-specific probes (SP-A, Hs00831305_s1; SP-B, Hs01090658_g1; SP-C, Hs00161628_m1; SP-D, Hs01108490_m1; ABCA3, Hs00975530_m1). Data were analized using the 2^-ΔΔ^Ct method in which 2^-ΔΔ^Ct is the amount of target normalized to an endogenous reference gene (TBP, TATA Box Protein) and relative to the positive control (adult lung tissue) (ΔCt  =  Ct (test gene)-Ct (housekeeping gene); ΔΔCt  =  ΔCt (sample) – ΔCt (positive control).

### Real-time exocytosis monitoring assay

DMSCs and ATII-LCs differentiated for 5 days grown on 8-well chamber slides (Ibidi, Instrumentación y componentes, Zaragoza, Spain) were incubated with 50 nM LysoTracker Green DND-26 (LTG) (Invitrogen, Fisher Scientific, Madrid, Spain) for 20 min in DMEM and washed with buffered solution. Cells were mounted into a heated chamber (37°C) for live imaging. Exocytosis was stimulated with ATP and PMA and secreted surfactant was stained in the continuous presence of 4 µM FM 1–43 (Invitrogen, Fisher Scientific, Madrid, Spain) as previously described [38]. Images were collected on a LSM 510 Meta Confocal Microscope (Zeiss, Carl Zeiss AG, Jena, Germany) mounted on a Zeiss Axiovert 200 M inverted microscope using 1 to 5% Argon laser power (488 nm) and a Kalman filter to average two scans. Emitted light was collected by a META detector set to 497–508 and 604–754 nm for LTG and FM 1–43 emissions, respectively. Single plane (4 µm) and z-stack images (4 µm, z-step = 2 µm) of multiple focal planes (6 to 8) were obtained using a high numerical-aperture (NA = 1.4) 63x oil-immersion objective. Images were acquired at 1.024×1.024 or 2.048×2.048 pixel resolution with 1 pixel  = 140 or 70 nm, respectively. For image handling and z-stack projections we used the public domain software ImageJ v.1.32j (Wayne Rasband, National Institute of Health).

### Electron microscopy

Pelleted DMSCs and ATII-LCs as well as secreted material from ATII-LCs were fixed by incubation with glutaraldehyde for 6 h at 4°C and then postfixed with 1% osmium tetroxide (both from Sigma-Aldrich Química, Spain) for 1 h. Samples were dehydrated with increasing concentrations of acetone and embedded in Spurr resin (Polysciences, Warrington, PA, USA). Ultrathin sections of the resin embedded samples were stained with uranyl acetate and lead citrate, and observed with a JEOL JEM-1230 transmission electron microscope (Jeol, Peabody, MA, USA).

### Adsorption kinetics assay

Surfactant materials were stained with a trace of BODIPY-PC (Invitrogen, Fisher Scientific, Madrid, Spain), upon preincubation with the probe at 45°C for 1 h (probe/surfactant  = 1 mol/mol%). Experiments were performed at 37°C in transparent polystyrene 96-well microtiter plates using a FLUOstar Optima (BMG Labtech, Ortenberg, Germany) plate reader as previously described [39]. Briefly, wells were filled with a solution containing 5 mg/ml of the strongly light-absorbing agent Brilliant Black (BB) and labeled-surfactant samples (0.5 µg/well) were injected at the bottom of the wells. After orbital shaking, fluorescence coming from surface-adsorbed material was measured by the instrument, whereas fluorescence coming from non-adsorbed complexes was quenched by BB. To evaluate the inhibitory effect of plasma proteins on the adsorption of the surfactant materials, experiments were also performed in the presence of increasing concentrations of human serum.

### Statistical analysis

Data represent means ± standard deviation. Statistical differences between groups were analyzed using one-way ANOVA with the Holm-Sidak method for paired comparisons of means (SigmaPlot 11.0, Germany). Statistical significance was taken at p<0.05.

## Results

### Morphological features of ATII-LCs

**Figure 1:**
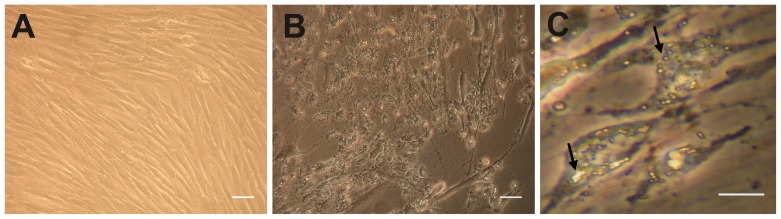
Morphology of DMSCs and ATII-LCs. Phase contrast images visualizing morphological changes of DMSCs during differentiation into ATII-LCs. *A)* Undifferentiated DMSCs showing a smooth and fibroblast-like morphology. *B)* Differentiated ATII-LCs after 4 days of incubation in differentiation medium, exhibiting a more flattened shape and a highly granulated cytoplasm. *C)* Magnified view of ATII-LCs displaying groups of highly-refractive cytoplasmic particles (arrows). Scale bars in *A* and *B* correspond to 100 µm and in *C* to 50 µm.

**Figure 2:**
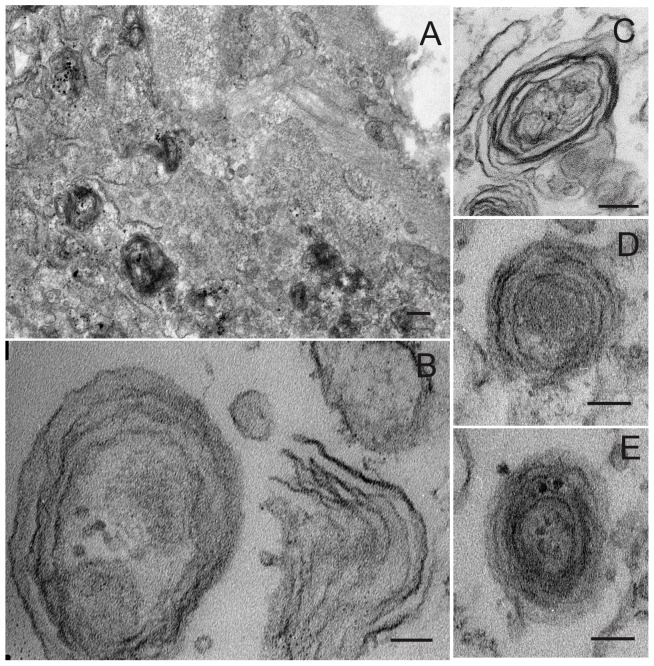
Ultrastructure of ATII-LCs. Transmission electron micrographs of differentiated ATII-LCs. *A)* Presence of numerous electron-dense cytoplasmic organelles and cell surface associated structures, probably microvilli. (x20,000; scale bar  = 200 nm). *B)* Membranous structures containing multiple concentric lamellae (left) and one partially unraveled fragmented lamellar body-like complex (right), displaying numerous unpacked membranes (x 120,000; scale bar  = 50 nm) *C*–*E)* Individual membranous structures with ellipsoid (C) or circular (D, E) shapes and a multilamellar ultrastructure (x 40000, scale bar  = 200 nm).

DMSCs are a homogeneous population of fibroblast-like cells with spindle-like shape and similar characteristics to bone marrow MSCs (Fig. 1A). After 4-5 days of culture in pulmonary differentiation medium, morphological changes are apparent such as a slightly more epithelioid shape (Fig. 1B, C) and the presence of light-dense cytoplasmic granules (Fig. 1C, arrows). The morphology of these dense particles in the cytoplasm of ATII-LCs was examined by transmission electron microscopy, revealing ellipsoid and circular membranous structures with multiple, variably densely packed concentric lamellae (Fig. 2A–E). These structures resemble the complex internal organization of the phospholipid-rich surfactant stores (lamellar bodies) in ATII cells, and are also similar in morphology to the lamellar body-like granules obtained from bronchoalveolar lavage and observed under the same conditions [40]. In addition, ATII-LCs exhibit at the culture-exposed cell side the presence of structures with the appearance of incipient microvilli (Fig. 2A), suggesting that the cells might be initiating a polarized morphological differentiation.

The differentiation of DMSCs to ATII-LCs showed high reproducibility. Many different placentas have been used and all of them produced cells that differentiated into ATII-LCs. Examination under the microscope revealed that in all cases near 100% of cells in the tissue culture plate developed the intracellular dense particles associated with lamellar body biogenesis, indicating very high differentiation yield.

### Synthesis of surfactant lipids and proteins

We then investigate whether the morphological differentiation of DMSCs into ATII-LCs, apparently implying the assembly of lamellar body-like structures, could be associated with activation of the synthesis of genuine surfactant phospholipid species, i.e. with the accumulation of disaturated phospholipids, and particularly of dipalmitoylphosphatidylcholine (DPPC). Table 1 summarizes data on the lipidomic analysis, by means of mass spectrometry, of the whole PC fraction from full membranes obtained from different pelleted cells (for the complet set of data regarding the content in single PC molecular species, see Table S1). Only 17,6% of PCs are disaturated species in membranes from non-differentiated DMSCs, with only around 10% of PC being DPPC. Upon differentiation, cells with the morphology of ATII-LCs apparently double their content in disaturated PC species up to 35%, with more than 25% being DPPC. Although the small number of samples analyzed precludes a proper statistical analysis of differences, it seems clear that the content in total disaturated PCs and DPPC of differentiated ATII-LCs approach that determined for a cell population enriched in true type II pneumocytes, as purified from lung tissue.

**Table 1:** Fraction of saturated phosphatidylcholines in undifferentiated (DMSCs) and differentiated (ATII-LCs) decidua-derived mesenchimal cells in comparison with primary ATII pneumocytes.

		Cell types	
	DMSCs (n = 2)	ATII-LCs (n = 3)	ATII
% DPPC	10,2±2,8	?(???)?25,4±9,3	26,4
% PC disat.	17,6±3,6	35,5±12,3	40,3

mean values ± s.d. have been calculated with respect to total PC.

**Figure 3:**
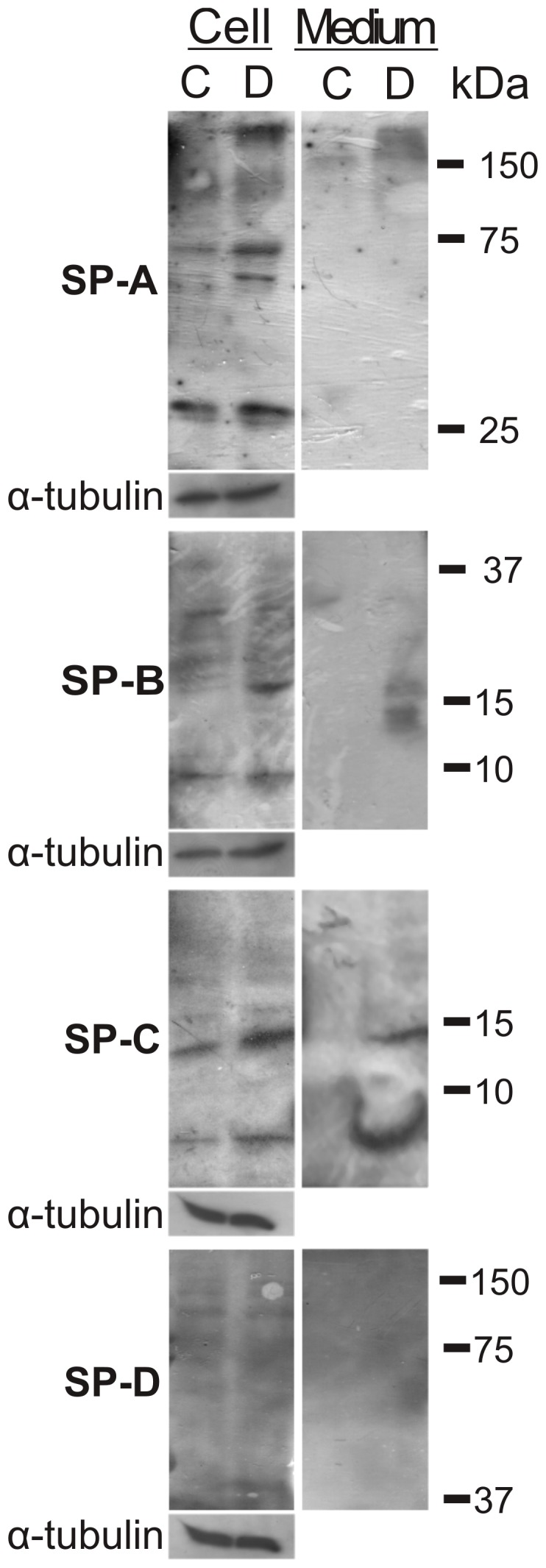
Pulmonary surfactant proteins in ATII-LC cultures. Western blots of pulmonary surfactant proteins (SP-A to SP-D) in DMSCs and ATII-LCs as well as the secreted proteins in the respective cell culture supernatants. The figure compares representative western blots (N = 3) after loading equivalent amounts of total protein, using α-tubulin expression as a loading control. (C =  control DMSCs; D =  differentiated ATII-LCs).

To determine whether ATII-LCs also synthesize, process and secrete surfactant proteins into the extracellular medium, the expression of these proteins was evaluated by western blot in both, DMSCs and 4-day differentiated ATII-LCs, as well as in their respective incubation media (Fig. 3). Enhanced levels of surfactant proteins SP-A, SP-B and SP-C were observed in ATII-LCs compared to DMSCs. Increased expression of trimeric (≈78 kDa), and particularly multimeric forms of SP-A (>150 kDa) can be discerned in ATII-LCs, likely as a consequence of only partial reduction of disulfides in the proteins embedded into the large secreted lipid-protein complexes. Presence of monomeric (≈8.7 kDa) and dimeric (≈17.4 kDa) forms of SP-B as well as monomeric (≈3.7 kDa) forms of SP-C are also more noticeable in the case of differentiated cells. Protein bands corresponding to molecular weights compatible with the presence of precursors of SP-B (proSP-B, ≈43 kDa) and SP-C (proSP-C, ≈21 kDa) as well as other intermediaries can be also distinguishable. Expression of SP-D was barely detectable. Presence of the mature forms of these proteins was also evident in the culture medium of ATII-LCs compared to DMSCs, suggesting that ATII-LCs were metabolically active and secreted these proteins. Basal expression of all the surfactant proteins was noticed in DMSCs, particularly in the case of SP-A and SP-C.

### Time-course of surfactant-related gene expression

**Figure 4:**
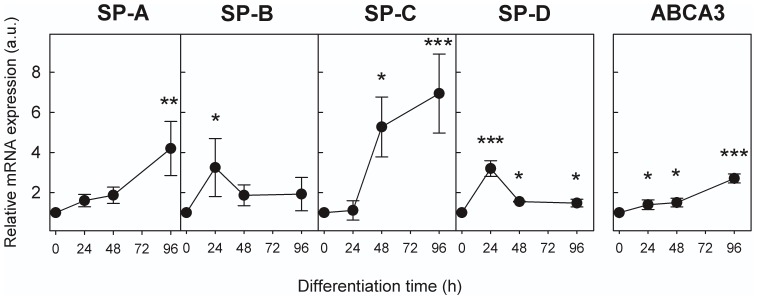
Expression of pulmonary surfactant-related genes during ATII-LC differentiation. Gene expression of surfactant proteins SP-A to SP-D and the lipid transporter ABCA3 during differentiation of DMSCs as determined by quantitative real-time PCR. Values are expressed as means (N = 5) of fold induction with respect to mRNA levels in DMSCs. Error bars indicate standard deviation. Human adult lung was used as a calibration control. The results were normalized to TBP values used as a housekeeping gene. * p<0.05), ** p<0.01, *** p<0.001.

To evaluate whether the expression of surfactant-related genes is regulated throughout differentiation, we assessed the presence of specific mRNAs in DMSCs and in ATII-LCs after various incubation times in the differentiation medium (24, 48 and 96 h) by quantitative real-time PCR (Fig. 4). SP-A mRNA levels were found to be significantly increased (p<0.001) 4-fold with respect to the gene expression level of DMSCs, upon 96 h of differentiation (Fig. 4). Similarly, SP-C mRNA expression showed a marked increase during differentiation, reaching levels of 7-fold induction at 96 h (p<0.001). However, the maximum increase in the level of gene expression for the proteins SP-B (p<0.05) and SP-D (p<0.001) took place at the beginning of differentiation, reaching values around 3-fold of induction at 24 h. The expression of these two genes decreased at 48 h and this level was maintained until 96 h.

We also examined the gene expression pattern of the surfactant lipid importer ABCA3, which plays a critical role in the biogenesis of surfactant complexes in ATII cells. ABCA3 gene expression level was 3-fold (p<0.001) increased from 24 h to 96 h of PLC differentiation.

### Surfactant exocytosis

**Figure 5:**
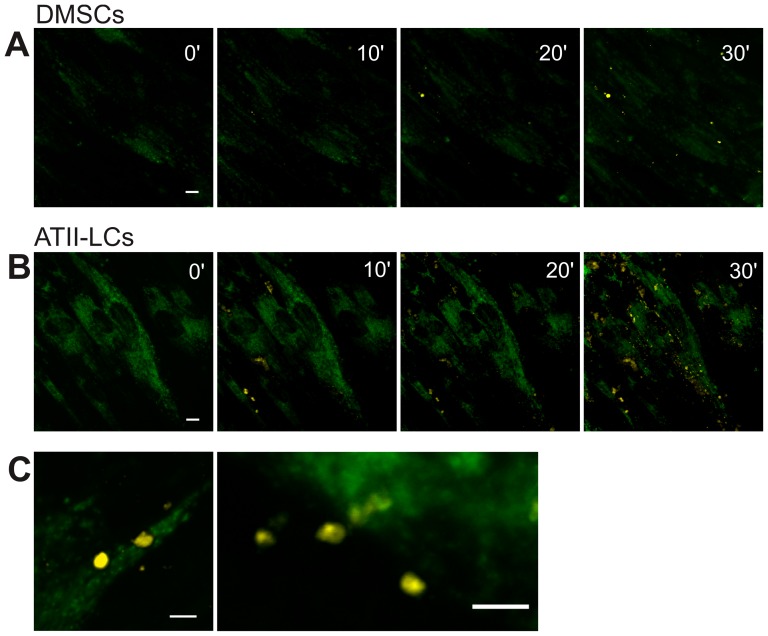
Secretion of pulmonary surfactant-like complexes by ATII-LCs. Confocal microscopy of DMSCs *(A)* and ATII-LCs *(B, C)* during stimulated exocytosis. Images are z-stack projections of multiple focal planes. Non-stimulated DMSCs and ATII-LCs were preincubated with LTG, showing green fluorescence originating from acidic compartments (e.g. lysosomes, late endosomes and Lamellar Bodies). Yellow fluorescence denotes FM 1–43 fluorescence, a lipid sensing probe. FM 1–43 was added to the cultures together with the secretagoges ATP and PMA immediately after time 0. Upon fusion of lipid-charged vesicles with the plasma membrane, bright yellow fluorescence resulted from FM 1–43 incorporation into secreted surfactant-like complexes. *(C)* Representative images of single confocal sections evidencing a high amount of secreted lipid assemblies associated to some ATII-LCs. Images were slightly corrected for a small loss in LTG fluorescence due to photobleaching. (Scale bar =  10 µm).

Since regulated secretion of lung surfactant is considered to be the most important functional characteristic in ATII cells, we aimed to explore the ability of ATII-LCs to exocytose their surfactant-like stores upon stimulation. For this purpose, we used a method previously described to monitor the exocytotic activity of primary cultures of ATII cells, taking advantage of the special properties of the fluorescent probes LTG and FM 1–43. LTG permeates through the plasma membrane and accumulates into acidic compartments such as surfactant-accumulating lamellar bodies and lysosomes. Thus, LTG was visualized as bright green fluorescence not only in ATII-LCs but also in DMSCs (Fig. 5). We stained cells with LTG to firstly demonstrate the presence of acidic compartments, secondly to visualize the cells and to get the LBs in the focal plane, and lastly to make sure that always approximately the same amount of LTG-positive vesicles and cells are in the respective field of measurement. The cells were then stimulated with ATP (100 µM) and PMA (100 nM), which are secretagogues in native ATII cells. At this point, FM 1–43, a membrane-impermeable dye that has no fluorescence in aqueous solution but exhibits fluorescence once intercalated into lipid membranes, was added to the cells. FM 1–43 enters through the fusion pore of the exocytosed vesicles, staining their lipid content and thus emitting a conspicuous yellow fluorescence. Stimulation of ATII-LCs cultures resulted in a progressive appearance of secreted lipid material visualized as bright yellow spots forming clusters at the periphery of the cells. In contrast, DMSCs were practically unresponsive to secretagogues. As a negative control, nonstimulated cells incubated in the presence of FM1–43 but in the absence of secretagogues showed no fluorescent response (data not shown).

**Figure 6:**
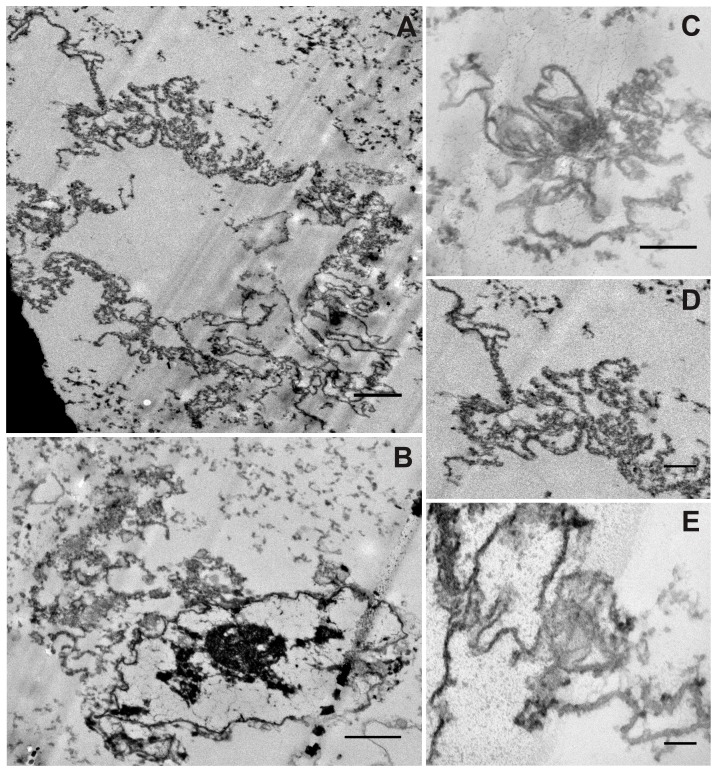
Ultrastructure of membrane complexes secreted by ATII-LCs. Transmission electron micrographs show harvested membranous complexes exocytosed by ATII-LCs upon stimulation with secretagoges for 6 h at 37°C. *A, D)* Multiple lipid membranes with numerous kinks constituting intricate structures possibly due to the presence of membrane-associated proteins. *B, C, E)* Membranous assemblies with some highly electron-dense areas, resembling the appearance of secreted and partially unraveled lamellar body-like particles. (Scale bars correspond to 1.000 nm in *A* and B, to 500 nm in *C* and *D* and 200 nm in *E*).

To further investigate the ultrastructural features of the lipid complexes secreted by the ATII-LCs, exocytosed material harvested from the extracellular medium was examined by transmission electron microscopy (Fig. 6). We discerned the presence of partially disorganized lamellar particles (Fig. 6B and C), as well as apparently unraveled lipid membranes. These membranes frequently showed the presence of defined structures, with marked kinks at some areas, suggesting the presence of membrane-associated proteins imposing significant perturbations (Fig. 6A, D and E). In the case of DMSCs, the scarce amount of secreted material harvested upon stimulation made impossible its structural examination.

### Biophysical activity of secreted surfactant-like material

**Figure 7:**
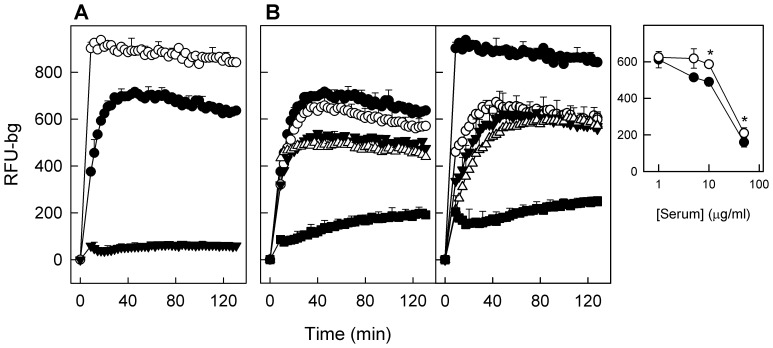
Interfacial adsorption properties of pulmonary surfactant-like complexes secreted by ATII-LCs. *A)* Comparison of surface adsorption properties of surfactant-like assemblies secreted by stimulated ATII-LCs (open circles) and secreted material by DMSCs (closed triangles) with respect to native surfactant purified from BAL (closed circles). Formation of a stably surface-associated lipid film is monitored by collecting BODIPY-fluorescence from the top of 96-wells during constant orbital shaking as described in detail in (38). Values of fluorescence were corrected for background (basal fluorescence prior to injection of labeled samples) and plotted as the mean of 5 experiments (N = 5) ± standard deviation. *B)* Dose-dependent inhibitory effect of human serum proteins on the adsorption kinetics of Native Surfactant (NS, left panel) and secreted material from ATII-LCs (SM, right panel) Serum protein concentrations were 0 (•), 1 (○), 5 (▾) 10 (▵) and 50 (▪) µg/ml. *Insert)* Fluorescence values taken after 60 min of adsorption of SM from ATII-LCs (open circles) and NS (closed circles) as a function of serum protein concentration. Adsorption efficacy in terms of amount of adsorbed material into the surface was significantly (p<0.05) higher in the case of SM than in NS at elevated serum concentrations (*). (RFU – bg  =  Background-substracted relative fluorescent units).

One of the most important properties of a functional surfactant is its capability to adsorb and accumulate rapidly and efficiently at the air-liquid interface, to form surface active films. To evaluate the interfacial adsorption properties of the material secreted by ATII-LCs, we have used a method developed in our laboratory that measures the kinetics of accumulation of surfactant at the interface and the resistance of the formed surface films and associated structures against mechanical agitation [39]. Surfactant-like complexes exocytosed by ATII-LCs displayed a rapid adsorption into the air-water interface. This adsorption was even faster than that of natural surfactant purified from bronchoalveolar lavages (BAL), which is typically used as standard material and a reference for optimal surfactant function. The scarce secreted material from stimulated DMSCs showed almost no adsorption properties, displaying a negligible accumulation at the interface (Fig. 7A).

Lung surfactant is, under some circumstances, susceptible to inhibition by substances (serum proteins, for instance) leaking from the blood into the airspaces in cases of a breakdown of the alveolar-capillary barrier function during e.g. high altitude pulmonary edema formation. We have compared the inhibitory effect of serum on the adsorption rate of both natural surfactant purified from BAL and material secreted by ATII-LCs (Fig. 7B). Surfactant-like material secreted by ATII-LCs showed a noticeable resistance to serum inhibition, displaying significantly better adsorption kinetics than native surfactant membranes, particularly in the presence of the highest concentrations of serum (p<0.05) (Fig. 7B).

## Discussion

We have previously described a population of adult mesenchymal stem cells isolated from the maternal portion of human extraembryonic membranes (Decidua-derived Mesenchymal Stem Cells, DMSCs) capable to differentiate into derivatives of all germ layers including ATII-like cells [33]. The present work demonstrates that placenta-derived Alveolar Type II-Like Cells (ATII-LCs) not only differentiate towards an ATII cell-like phenotype but develop into truly functional units with the potential of expressing surfactant-related genes and proteins and with the capability of producing, storing and secreting highly surface-active, lipid-based assemblies into the extracellular fluid, thereby mimicking the exocytotic behavior of native ATII cells [41].

Examination by light and transmission electron microscopy reveals that the morphology of ATII-LCs is consistent with the ATII cell phenotype, including lamellar body-like structures similar to those observed in material obtained from bronchoalveolar lavage [40], and a cell shape suggesting incipient formation of microvilli in contact with the aqueous phase. Preliminary experiments extending the culture of these differentiated cells into air-liquid interfaces reveal further morphological differentiation into cuboidal shapes, with conspicuous microvilli and a clear resemblance to true alveolar type II morphologies (not shown). The characterization of such a differentiation at the physiologically meaningful context of a true air-exposed epithelium, including the potential acquisition of a truly polarized morphology, is beyond the scope of the current work, but confirms that the predifferentiation stage described here is relevant in terms of pneumocyte-like differentiation. On the other hand, ATII-LCs were shown to overexpress mRNAs encoding surfactant proteins A-D and ABCA3 in response to the differentiation signals. Noticeably, mRNA levels of SP-B and SP-D were upregulated at the onset of the differentiation process (early expression genes) whereas mRNA levels of SP-A and SP-C were increased later (late expression genes). The biological relevance of this distinct expression pattern is unclear but will be explored in subsequent studies. Previous studies reported an earlier expression of the SP-B gene but also that of SP-C, previous to the expression of SP-A, in human fetuses, suggesting an independent regulation of hydrophobic surfactant proteins and SP-A during lung development [42]–[44]. On the other hand, the expression of mRNAs encoding surfactant proteins and ABCA3 has been reported as being collectively regulated by a number of transcription factors like the thyroid transcription factor-1 (TTF-1) and the Forkhead ortholog a2 (Foxa2) [45], [46] though it seems that their expression in the lung is regulated by even more complex mechanisms [47]. The particular signaling pathways involved in the differentiation of DMSCs into the lung phenotype as it takes place under our culture conditions remains as an open question that will need further investigation but that was not the purpose of the present work. Our results indicate an initial differentiation towards a surfactant producing ATII-LC phenotype, but one should not discard that patterns of protein expression at longer times could be related with further differentiation into other phenotypes such as those typical of type I pneumocytes. Differentiation of ATII cells of pulmonary origin into ATI cells is widely documented both in vitro and in vivo [48], [49]. In our experiments, the onset for enhanced expression of all these mRNAs correlated with the appearance of lamellar body-like intracellular particles, an evident signal of lung maturation [50]–[52]. Surprisingly, DMSCs were shown to already express basal levels of surfactant proteins. This could be related to their placental origin. Presence of SP-A and SP-D has been previously reported in human amniotic epithelium and chorio-decidual layers [53], [54]. As a matter of fact, SP-A has been proposed to participate as a factor inducing parturition [54]. In addition, SP-A and SP-D lectin domains have been described to display an active role in the recognition and clearance of pathogens from amniotic fluid [53]. Presence of SP-B and SP-C has also been demonstrated in placenta and other fetal environments [55], [56]. Although their role is still under debate, it has been suggested that SP-C might participate in modulating inflammatory responses in the extraembyonic environment [55]. Our results demonstrate that differentiated ATII-LCs have the capability to properly process surfactant proteins from their precursors into the mature forms, a processing that has been specifically linked with the ATII phenotype, but that should likely be extended to other epithelia related with the fetal/environment interface. Interestingly, the amount of surfactant proteins found in the extracellular fluid of ATII-LC cultures was also higher than that in the medium of DMSCs, which is suggestive of a functional and metabolically active state of ATII-LCs.

The major function of ATII cells is the production and secretion of surfactant. The fact that ATII-LCs are able to synthetise and accumulate disaturated phospholipid species and to secrete functional membranous lipid complexes upon stimulation with secretagogues further supports the differentiated state of ATII-LCs and their pneumocyte-like functionality. We have implemented a method to real-time visualizing single events of surfactant release, previously described and validated for native ATII cell primary cultures [35], [38], [57]–[59]. Despite the fact that LTG staining was not specifically limited to LBs due to the great amount of lysosome-related small vesicles also present in DMSCs, ATII-LCs displayed much more acidic compartments (probably LBs) than DMSCs and only ATII-LCs were clearly responsive to the addition of secretagogues. Upon incubating with ATP and PMA, ATII-LCs exocytosed an appreciable number of lipid vesicles stained with FM1-43 as discrete spherical fluorescent spots. These observations indicate that the secreted material remains in an aggregated state and does not readily disperse into the buffer solution as it has been also described for material secreted by ATII cell primary cultures [38]. Interestingly, the time course of exocytosis in ATII-LCs displayed a slow rate, mimicking the secretory behavior well documented in rat ATII cell primary cultures [38], [57]. As revealed by transmission electron microscopy, surfactant-like material secreted from ATII-LCs contained a mixture of different structures, mainly partially unpacked osmiophilic entities as well as multiple lipid membrane patches with particular morphologies, probably residual features from a previous more complex organization. In general terms, the structures observed resemble the lipoprotein complexes exocytosed upon stimulation by native ATII cells [40], and had similar efficient interfacial adsorption capabilities. Previous work had demonstrated that surfactant complexes freshly secreted by primary cultures of type II pneumocytes adsorb to the interface at higher rates than surfactant complexes isolated from bronchoalveolar lavage [39]. This has been interpreted considering that material obtained from airspaces could actually contain not only freshly secreted surfactant but also surfactant complexes that may have been already somehow spent, or partly inactivated due to oxidation, or to insertion of spurious components. The fact that material secreted by ATII-LCs also shows superior interfacial adsorption than surfactant purified from lungs argues in favour of their true functional pneumocyte-like phenotype. Pulmonary surfactant-like assemblies secreted by ATII-LCs even displayed a higher resistance to inhibition by serum than whole native surfactant purified from BAL. The limited amounts of material available has prevented to assess whether the interfacial films formed by the material produced by ATII-LCs are also competent to reach and sustain very low tensions under compression-expansion dynamics. Future optimization and scaling-up of conditions for cell culture, differentiation and secreted surfactant harvesting will allow to evaluate not only the full performance of this surfactant but the potential of this strategy to approach the biotechnological production, at large scale, of humanized clinical surfactants for therapeutic applications.

Under the differentiation conditions used here, the cultures appear as highly enriched in ATII-LCs, exhibiting remarkable yields of differentiation as previously assessed in immunocytochemical stainings for proSP-C [30]. ATII-LCs display a dynamic phenotype throughout the differentiation process from initial immature phases to mature functional stages. As primary cultures and once differentiated, ATII-LCs cannot be expanded further in culture. However and unlike other placenta-derived MSCs from fetal membranes (amnion and chorion) and chorionic villi [60]–[62] DMSCs exhibit a notably high life span and subsequently, large amount of cells can be obtained providing with a virtually unlimited source to generate derived ATII-LCs. In spite that our differentiation procedure has shown to be effective, simple and quick, we presume that ATII-LCs are still in a less differentiated state than true ATII cells as seen *in situ* in the lung epithelium or in isolated primary cultures. We speculate that the pneumocyte-like phenotype of ATII-LCs could progress further to full differentiation upon immersion into a more complete lung environment, either in the tissue *in vivo* or in more elaborated cell culture models that could incorporate features like the presence of an air-liquid interface. In this sense, Transwell devices have been reported as successful for lung differentiation by other groups [24], [26], and it could provide additional signaling to improve full differentiation of ATII-LCs.

The potential of DMSCs to differentiate into pneumocyte-like phenotypes had been previously reported by our group, but the present findings offer novel insights in the field since this is, to our knowledge, the first report of a population of lung-derived stem cells capable of secreting a lipid-rich active pulmonary surfactant-like material into the air-liquid interface. In addition, the multipotent adult DMSCs used here display important advantages such as a high availability, since placental tissue is a virtually unrestricted material, its use does not evoke ethical concerns, and cells are easily isolated yielding a high amount of material. DMSCs can be cultured and multiplied at a certain scale, and this could open a novel way to harvest freshly secreted surfactant complexes for further investigations.

Clinically, administration of MSCs from different origins into animal models of lung injury has been demonstrated by a number of authors to be a valuable therapeutic tool [24], [26], [63]–[68]. We have recently reported that DMSCs could be useful for future clinical applications [33], [69]. DMSCs subjected to hepatic differentiation conditions have shown remarkable functional capabilities, suitable to be used as models for studying liver physiology as well as liver diseases with a potential application in hepatic regeneration [33]. On the other hand, we have also demonstrated the migration and engraftment of DMSCs into mammary tumors and the inhibitory effect of the cells on the growth of primary tumors and in the development of new tumors [69]. DMSCs do not express the major histocompatibility complex class II and the T cell costimulatory molecules, conferring them an intrinsically hypoimmunogenic and immunomodulating stem cell character [30]. DMSCs or ATII-LCs could therefore open new strategies as a potential therapeutic agent for the treatment of lung injury.

In conclusion, the results reported in this work provide some support for considering ATII-LCs as appropriate candidates for *ex vivo* ATII cell models, with the possibility of introducing genetic modifications to study surfactant biogenesis, in particular protein and lipid synthesis, maturation, and traffic, as well as the packing and storage of surfactant lipid-protein complexes into LBs.

## Supporting Information

Table S1 (DOCX)
