# Supplementary material for: Human Decidua-Derived Mesenchymal Stem Cells Differentiate into Functional Alveolar Type II-Like Cells that Synthesize and Secrete Pulmonary Surfactant Complexes
Source: PLoS One. 2014 Oct 15;9(10):e110195. doi: 10.1371/journal.pone.0110195 (PMC4198213; doi:10.1371/journal.pone.0110195)
Supplement: Table S1 — (DOCX) [file pone.0110195.s001.docx]

**Table S1**

**Total fraction of phosphatidylcholine molecular species in undifferentiated (DMSCs) and differentiated (ATII-LCs) decidua-derived mesenchimal cells in comparison with primary ATII pneumocytes**

|  | **Cell types** | | | | |  |
| --- | --- | --- | --- | --- | --- | --- |
| **PC^*^**  **molecular species** | **DMSCs**  **(n=2)** | | **ATII-LCs (n=3)** | **ATII** | |  |
| **28:0-PC** | 0.5 ± 0.01 |  | 0.2 ± 0.1 | 0.5 |  |  |
| **30:0-PC** | 5.0 ± 0.4 |  | 6.8 ± 2.7 | 10.5 |  |  |
| **30:1-PC** | 1.6 ± 0.2 |  | 0.5 ± 0.1 | 1.1 |  |  |
| **32:0-PC** | 10.2 ± 2.8 |  | 25.4 ± 9.3 | 26.4 |  |  |
| **32:1-PC** | 13.7 ± 0.5 |  | 10.0 ± 0.9 | 16.3 |  |  |
| **32:2-PC** | 2.7 ± 0.3 |  | 0.8 ± 0.2 | 2.2 |  |  |
| **34:0-PC** | 1.8 ± 0.4 |  | 3.1 ± 0.3 | 2.8 |  |  |
| **34:1-PC** | 21.8 ± 0.3 |  | 23.0 ± 3.1 | 9.9 |  |  |
| **34:2-PC** | 11.1 ± 1.3 |  | 5.6 ± 1.2 | 8.8 |  |  |
| **34:3-PC** | 1.1 ± 0.1 |  | 0.4 ± 0.1 | 1.5 |  |  |
| **34:4-PC** | 0.1 ± 0.01 |  | - | 0.4 |  |  |
| **36:0-PC** | - |  | - | 0.1 |  |  |
| **36:1-PC** | 5.8 ± 0.1 |  | 7.0 ± 2.5 | 0.5 |  |  |
| **36:2-PC** | 16.0 ± 0.4 |  | 9.6 ± 3.9 | 2.3 |  |  |
| **36:3-PC** | - |  | 1.4 ± 0.4 | 2.0 |  |  |
| **36:4-PC** | 1.1 ± 0.1 |  | 0.9 ± 0.2 | 7.4 |  |  |
| **36:5-PC** | 0.3 ± 0.02 |  | 0.2 ± 0.1 | 0.8 |  |  |
| **38:2-PC** | 1.2 ± 0.1 |  | 0.6 ± 0.4 | 0.1 |  |  |
| **38:3-PC** | 1.8 ± 0.1 |  | 1.1 ± 0.8 | 0.6 |  |  |
| **38:4-PC** | 1.0 ± 0.1 |  | 1.0 ± 0.4 | 2.6 |  |  |
| **38:5-PC** | 1.3 ± 0.1 |  | 1.1 ± 0.5 | 1.0 |  |  |
| **38:6-PC** | 0.6 ± 0.1 |  | 0.5 ± 0.1 | 1.2 |  |  |
| **40:5-PC** | 0.6 ± 0.1 |  | 0.4 ± 0.3 | 0.3 |  |  |
| **40:6-PC** | 0.4 ± 0.1 |  | 0.2 ± 0.2 | 0.1 |  |  |
| **40:6-PC** | 0.2 ± 0.01 |  | 0.2 ± 0.1 | 0.3 |  |  |
| ^*^The different molecular species are identified as **n:m-PC**, where **n** is the total number of carbons of the *sn-1* and *sn-2* acyl chains, and **m**, the total number of double bonds  mean values ± s.d. have been calculated as percent fraction with respect to total PC | | | | | |  |
